# 4-Methyl­pyridinium 2-carb­oxy-4,5-dichloro­benzoate monohydrate

**DOI:** 10.1107/S1600536810015503

**Published:** 2010-05-08

**Authors:** Graham Smith, Urs D. Wermuth

**Affiliations:** a Faculty of Science and Technology, Queensland University of Technology, GPO Box 2434, Brisbane, Queensland 4001, Australia

## Abstract

In the structure of the 1:1 proton-transfer compound of 4-methyl­pyridine (γ-picoline) with 4,5-dichloro­phthalic acid, C_6_H_8_N^+^·C_8_H_3_Cl_2_O_4_^−^·H_2_O, determined at 200 K, the 4,5-dichloro­phthalate anions are bridged by the water mol­ecule through O—H⋯O_carbox­yl_ hydrogen bonds, giving zigzag chains which extend along the *c*-axis direction. The 4-methyl­pyridinium cations are linked to the chains through single N—H⋯O_water_ hydrogen bonds and occupy the voids within the chains in the one-dimensional structure. The anions have the common ‘planar’ conformation with a short intra­molecular O—H⋯O_carbox­yl_ hydrogen bond.

## Related literature

For the structures of other hydrogen 4,5-dichloro­phthalate salts, see: Mallinson *et al.* (2003 ▶); Bozkurt *et al.* (2006 ▶); Smith *et al.* (2007 ▶, 2008*a* ▶,*b* ▶, 2009 ▶, 2009*a* ▶,*b* ▶); Smith & Wermuth (2010*a* ▶,*b* ▶).
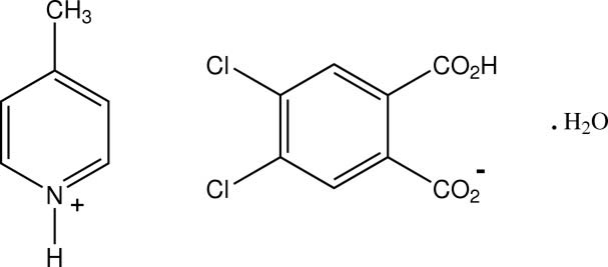


## Experimental

### 

#### Crystal data

C_6_H_8_N^+^·C_8_H_3_Cl_2_O_4_^−^·H_2_O
*M* *_r_* = 346.15
Monoclinic, 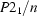

*a* = 3.8398 (3) Å
*b* = 29.5531 (17) Å
*c* = 12.9855 (7) Å
β = 90.054 (6)°
*V* = 1473.57 (16) Å^3^
*Z* = 4
Mo *K*α radiation
μ = 0.46 mm^−1^
*T* = 200 K
0.30 × 0.20 × 0.08 mm

#### Data collection

Oxford Diffraction Gemini-S CCD-detector diffractometer
Absorption correction: multi-scan (*SADABS*; Sheldrick, 1996 ▶) *T*_min_ = 0.930, *T*_max_ = 0.980
8898 measured reflections
2579 independent reflections
2156 reflections with *I* > 2σ(*I*)
*R*_int_ = 0.021

#### Refinement

*R*[*F* ^2^ > 2σ(*F* ^2^)] = 0.045
*wR*(*F* ^2^) = 0.129
*S* = 1.29
2579 reflections
215 parameters
H atoms treated by a mixture of independent and constrained refinement
Δρ_max_ = 0.24 e Å^−3^
Δρ_min_ = −0.33 e Å^−3^

### 

Data collection: *CrysAlis PRO* (Oxford Diffraction, 2009 ▶); cell refinement: *CrysAlis PRO*; data reduction: *CrysAlis PRO*; program(s) used to solve structure: *SIR92* (Altomare *et al.*, 1994 ▶); program(s) used to refine structure: *SHELXL97* (Sheldrick, 2008 ▶) within *WinGX* (Farrugia, 1999 ▶); molecular graphics: *PLATON* (Spek, 2009 ▶); software used to prepare material for publication: *PLATON*.

## Supplementary Material

Crystal structure: contains datablocks global, I. DOI: 10.1107/S1600536810015503/jj2029sup1.cif

Structure factors: contains datablocks I. DOI: 10.1107/S1600536810015503/jj2029Isup2.hkl

Additional supplementary materials:  crystallographic information; 3D view; checkCIF report

## Figures and Tables

**Table 1:** Hydrogen-bond geometry (Å, °)

*D*—H⋯*A*	*D*—H	H⋯*A*	*D*⋯*A*	*D*—H⋯*A*
N1*A*—H1*A*⋯O1*W*	0.85 (5)	1.82 (5)	2.663 (5)	170 (5)
O1*W*—H11*W*⋯O21	0.79 (5)	2.02 (5)	2.793 (4)	168 (5)
O1*W*—H12*W*⋯O11^i^	0.78 (5)	2.03 (5)	2.806 (4)	170 (4)
O21—H21⋯O12	1.00 (6)	1.38 (6)	2.376 (4)	180 (8)

Symmetry code: (i) 
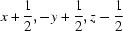
.

**Fig. 1.**
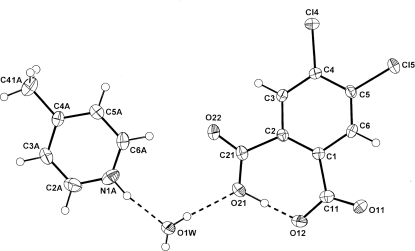
Molecular configuration and atom numbering scheme for the 4-methylpyridinium cation, the hydrogen 4,5-dichlorophthalate anion and the water molecule of hydration (O1W) in (I). Non-H atoms are shown as 50% probability displacement ellipsoids with inter-species hydrogen bonds shown as dashed lines.

**Fig. 2.**
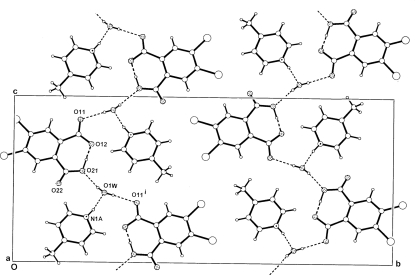
The one-dimensional hydrogen-bonded chain structures extending down the c direction in the unit cell of (I). For symmetry code see Table 1.

## supplementary crystallographic information

### Comment

The 1:1 proton-transfer compounds of 4,5-dichlorophthalic acid (DCPA) with the nitrogen Lewis bases commonly have low-dimensional hydrogen-bonded structures (Mallinson *et al.*, 2003; Bozkurt *et al.*, 2006; Smith *et al.*, 2007, 2008a, 2008b, 2009a, 2009b; Smith *et al.*,  2009; Smith & Wermuth, 2010a, 2010b). With the majority of these structures, e.g. the brucinium salt (Smith *et al.*, 2007), the DCPA anions are essentially planar (the 'planar' conformation) with short intramolecular carboxylic acid O–H···O_carboxyl_ hydrogen bonds. These features were also found in the structure of the hydrated 1:1 proton-transfer compound of DCPA with 4-methylpyridine (γ-picoline), the title compound C_6_H_8_N^+^ C_8_H_3_Cl_2_O_4_^-^ . H_2_O (I), reported here.

In (I) (Fig. 1), the 4,5-dichlorophthalate anions are bridged by the water molecule through O–H···O_carboxyl_ hydrogen bonds giving zig-zag chains which extend along the *c* axial direction of the unit cell (Fig. 2). The 4-methylpyridine cations are linked to the chains through single N–H···O_water_ hydrogen bonds and occupy the voids formed within the chains, in the one-dimensional structure. There are no cation–anion π–π ring stacking interactions such as are present in some of the DCPA compounds.

The DCPA anion has the 'planar' conformation [torsion angles C2–C1–C11–O11, -173.2 (3)°: C1–C2–C21–O22, 169.9 (3)°], with the short intramolecular O–H···O_carboxyl_ hydrogen bond [2.376 (4) Å]. Associated with this bond is a significant distortion of the *exo*-C1 and C2 bond angles [C1–C2–C21, 128.9 (3)° and C2–C1–C11, 128.8 (3)°]. This and a lengthening of the C1–C11 and C2–C21 bonds [1.527 (5) and 1.531 (5) Å] are features inherent in the 'planar' DCPA anions in the overall series of 1:1 proton-transfer compounds.

### Experimental

The title compound (I) was synthesized by heating together for 10 min under reflux 1 mmol quantities of γ-picoline and 4,5-dichlorophthalic acid in 50 ml of methanol. The product after complete room-temperature evaporation of the hot-filtered solution was microcrystalline. Recrystallization from water gave colourless flat needles (m.p. 445 K) from which a specimen suitable for X-ray analysis was cleaved.

### Refinement

Hydrogen atoms potentially involved in hydrogen-bonding interactions were located by difference methods and their positional and isotropic displacement parameters were refined. Other H atoms were included in the refinement at calculated positions [C–H_aromatic_ = 0.93 Å and C–H_aliphatic_ = 0.98 Å] and treated as riding models with *U*_iso_(H) = 1.2*U*_eq_(C).

### Crystal data

**Table:**

C_6_H_8_N^+^·C_8_H_3_Cl_2_O_4_^−^·H_2_O	*F*(000) = 712
*M**_r_* = 346.15	*D*_x_ = 1.560 Mg m^−^^3^
Monoclinic, *P*2_1_/*n*	Melting point: 445 K
Hall symbol: -P 2yn	Mo *K*α radiation, λ = 0.71073 Å
*a* = 3.8398 (3) Å	Cell parameters from 4516 reflections
*b* = 29.5531 (17) Å	θ = 3.1–28.7°
*c* = 12.9855 (7) Å	µ = 0.46 mm^−^^1^
β = 90.054 (6)°	*T* = 200 K
*V* = 1473.57 (16) Å^3^	Plate, colourless
*Z* = 4	0.30 × 0.20 × 0.08 mm

### Data collection

**Table:**

Oxford Diffraction Gemini-S CCD-detector diffractometer	2579 independent reflections
Radiation source: Enhance (Mo) X-ray source	2156 reflections with *I* > 2σ(*I*)
graphite	*R*_int_ = 0.021
Detector resolution: 16.08 pixels mm^-1^	θ_max_ = 25.0°, θ_min_ = 3.1°
ω scans	*h* = −4→4
Absorption correction: multi-scan (*SADABS*; Sheldrick, 1996)	*k* = −35→31
*T*_min_ = 0.930, *T*_max_ = 0.980	*l* = −15→15
8898 measured reflections	

### Refinement

**Table:**

Refinement on *F*^2^	Primary atom site location: structure-invariant direct methods
Least-squares matrix: full	Secondary atom site location: difference Fourier map
*R*[*F*^2^ > 2σ(*F*^2^)] = 0.045	Hydrogen site location: geom'
*wR*(*F*^2^) = 0.129	H atoms treated by a mixture of independent and constrained refinement
*S* = 1.29	*w* = 1/[σ^2^(*F*_o_^2^) + (0.042*P*)^2^ + 1.7305*P*] where *P* = (*F*_o_^2^ + 2*F*_c_^2^)/3
2579 reflections	(Δ/σ)_max_ < 0.001
215 parameters	Δρ_max_ = 0.24 e Å^−^^3^
0 restraints	Δρ_min_ = −0.33 e Å^−^^3^

### Special details

**Table:**

Geometry. Bond distances, angles etc. have been calculated using the rounded fractional coordinates. All su's are estimated from the variances of the (full) variance-covariance matrix. The cell esds are taken into account in the estimation of distances, angles and torsion angles
Refinement. Refinement on *F*^2^ for ALL reflections except those flagged by the user for potential systematic errors. Weighted *R*-factors wR and all goodnesses of fit *S* are based on *F*^2^, conventional *R*-factors *R* are based on *F*, with *F* set to zero for negative *F*^2^. The observed criterion of *F*^2^ > σ(*F*^2^) is used only for calculating -*R*-factor-obs etc. and is not relevant to the choice of reflections for refinement. *R*-factors based on *F*^2^ are statistically about twice as large as those based on *F*, and *R*-factors based on ALL data will be even larger.

### Fractional atomic coordinates and isotropic or equivalent isotropic displacement parameters (Å^2^)

**Table:**

	*x*	*y*	*z*	*U*_iso_*/*U*_eq_	
Cl4	0.1942 (3)	−0.02601 (3)	0.63754 (7)	0.0379 (3)	
Cl5	−0.1079 (2)	0.00515 (3)	0.85706 (7)	0.0372 (3)	
O11	0.0394 (8)	0.17470 (9)	0.8599 (2)	0.0471 (10)	
O12	0.2068 (8)	0.20432 (9)	0.7127 (2)	0.0490 (10)	
O21	0.4257 (8)	0.18118 (9)	0.5499 (2)	0.0504 (10)	
O22	0.6118 (7)	0.11959 (9)	0.47323 (19)	0.0399 (9)	
C1	0.1694 (8)	0.12249 (11)	0.7262 (2)	0.0233 (10)	
C2	0.3066 (8)	0.10876 (11)	0.6304 (2)	0.0225 (10)	
C3	0.3086 (8)	0.06268 (11)	0.6068 (2)	0.0249 (10)	
C4	0.1817 (8)	0.03024 (11)	0.6731 (3)	0.0252 (10)	
C5	0.0513 (8)	0.04386 (11)	0.7687 (2)	0.0252 (10)	
C6	0.0428 (8)	0.08917 (11)	0.7929 (2)	0.0254 (10)	
C11	0.1371 (9)	0.17020 (12)	0.7704 (3)	0.0328 (11)	
C21	0.4622 (9)	0.13795 (12)	0.5446 (3)	0.0291 (11)	
N1A	1.1032 (8)	0.19332 (11)	0.2708 (3)	0.0411 (11)	
C2A	1.2276 (11)	0.21031 (13)	0.1826 (3)	0.0447 (14)	
C3A	1.3735 (9)	0.18251 (13)	0.1103 (3)	0.0370 (12)	
C4A	1.3940 (8)	0.13645 (12)	0.1288 (2)	0.0282 (10)	
C5A	1.2671 (9)	0.12034 (12)	0.2211 (3)	0.0333 (11)	
C6A	1.1180 (9)	0.14896 (14)	0.2913 (3)	0.0369 (11)	
C41A	1.5391 (10)	0.10520 (15)	0.0494 (3)	0.0463 (14)	
O1W	0.7863 (9)	0.23983 (11)	0.4193 (2)	0.0462 (10)	
H3	0.39930	0.05350	0.54380	0.0300*	
H6	−0.05070	0.09800	0.85580	0.0300*	
H21	0.334 (15)	0.191 (2)	0.618 (5)	0.100 (19)*	
H1A	1.012 (12)	0.211 (2)	0.315 (4)	0.049 (10)*	
H2A	1.21470	0.24130	0.17040	0.0540*	
H3A	1.45850	0.19450	0.04900	0.0450*	
H5A	1.28280	0.08960	0.23580	0.0400*	
H6A	1.02810	0.13770	0.35270	0.0440*	
H41A	1.61710	0.12240	−0.00890	0.0560*	
H42A	1.36170	0.08430	0.02790	0.0560*	
H43A	1.73170	0.08870	0.07810	0.0560*	
H11W	0.666 (13)	0.2227 (18)	0.449 (4)	0.065 (17)*	
H12W	0.694 (11)	0.2626 (17)	0.405 (3)	0.046 (14)*	

### Atomic displacement parameters (Å^2^)

**Table:**

	*U*^11^	*U*^22^	*U*^33^	*U*^12^	*U*^13^	*U*^23^
Cl4	0.0516 (6)	0.0224 (4)	0.0396 (5)	−0.0018 (4)	0.0113 (4)	−0.0043 (4)
Cl5	0.0483 (5)	0.0316 (5)	0.0318 (5)	−0.0034 (4)	0.0108 (4)	0.0091 (4)
O11	0.074 (2)	0.0310 (15)	0.0363 (16)	−0.0029 (13)	0.0195 (14)	−0.0095 (12)
O12	0.084 (2)	0.0225 (14)	0.0406 (16)	0.0024 (13)	0.0133 (15)	0.0012 (12)
O21	0.083 (2)	0.0280 (15)	0.0403 (17)	−0.0001 (14)	0.0222 (15)	0.0098 (12)
O22	0.0534 (16)	0.0394 (15)	0.0269 (14)	0.0010 (12)	0.0122 (12)	0.0059 (11)
C1	0.0232 (17)	0.0233 (17)	0.0234 (17)	0.0009 (13)	−0.0027 (13)	0.0001 (13)
C2	0.0225 (16)	0.0244 (17)	0.0206 (17)	0.0021 (13)	−0.0019 (13)	0.0042 (13)
C3	0.0273 (17)	0.0264 (18)	0.0209 (16)	0.0014 (13)	0.0039 (13)	−0.0017 (13)
C4	0.0281 (17)	0.0212 (17)	0.0264 (17)	0.0023 (13)	0.0023 (13)	−0.0015 (13)
C5	0.0268 (17)	0.0249 (17)	0.0240 (17)	0.0002 (13)	0.0028 (13)	0.0057 (14)
C6	0.0256 (17)	0.0327 (19)	0.0179 (16)	0.0014 (14)	0.0017 (13)	−0.0036 (14)
C11	0.039 (2)	0.0253 (19)	0.034 (2)	0.0018 (15)	0.0015 (16)	−0.0026 (15)
C21	0.0315 (19)	0.032 (2)	0.0237 (18)	−0.0023 (14)	0.0016 (14)	0.0051 (15)
N1A	0.0374 (18)	0.043 (2)	0.043 (2)	0.0010 (14)	−0.0022 (15)	−0.0190 (16)
C2A	0.046 (2)	0.029 (2)	0.059 (3)	−0.0040 (17)	0.001 (2)	0.0014 (19)
C3A	0.037 (2)	0.040 (2)	0.034 (2)	−0.0036 (16)	0.0039 (16)	0.0114 (17)
C4A	0.0237 (17)	0.038 (2)	0.0228 (17)	−0.0011 (14)	−0.0019 (13)	−0.0018 (15)
C5A	0.0339 (19)	0.032 (2)	0.034 (2)	−0.0021 (15)	−0.0002 (16)	0.0050 (16)
C6A	0.036 (2)	0.050 (2)	0.0248 (19)	−0.0044 (17)	0.0016 (15)	0.0032 (17)
C41A	0.037 (2)	0.059 (3)	0.043 (2)	0.0042 (19)	0.0065 (18)	−0.013 (2)
O1W	0.078 (2)	0.0254 (16)	0.0352 (16)	0.0033 (16)	0.0159 (15)	0.0043 (13)

### Geometric parameters (Å, °)

**Table:**

Cl4—C4	1.726 (3)	C3—C4	1.378 (5)
Cl5—C5	1.732 (3)	C4—C5	1.398 (5)
O11—C11	1.229 (5)	C5—C6	1.376 (5)
O12—C11	1.285 (5)	C3—H3	0.9300
O21—C21	1.287 (4)	C6—H6	0.9300
O22—C21	1.218 (5)	C2A—C3A	1.368 (5)
O21—H21	1.00 (6)	C3A—C4A	1.385 (5)
O1W—H12W	0.78 (5)	C4A—C5A	1.379 (5)
O1W—H11W	0.79 (5)	C4A—C41A	1.493 (5)
N1A—C6A	1.339 (5)	C5A—C6A	1.369 (5)
N1A—C2A	1.339 (5)	C2A—H2A	0.9300
N1A—H1A	0.85 (5)	C3A—H3A	0.9300
C1—C11	1.527 (5)	C5A—H5A	0.9300
C1—C6	1.399 (4)	C6A—H6A	0.9300
C1—C2	1.411 (4)	C41A—H43A	0.9600
C2—C3	1.396 (5)	C41A—H41A	0.9600
C2—C21	1.531 (5)	C41A—H42A	0.9600
			
C21—O21—H21	112 (3)	C4—C3—H3	119.00
H11W—O1W—H12W	114 (5)	C2—C3—H3	119.00
C2A—N1A—C6A	121.5 (4)	C1—C6—H6	119.00
C2A—N1A—H1A	120 (4)	C5—C6—H6	119.00
C6A—N1A—H1A	119 (4)	N1A—C2A—C3A	120.6 (4)
C2—C1—C11	128.9 (3)	C2A—C3A—C4A	119.7 (3)
C6—C1—C11	112.9 (3)	C3A—C4A—C41A	120.7 (3)
C2—C1—C6	118.3 (3)	C3A—C4A—C5A	118.1 (3)
C1—C2—C21	128.8 (3)	C5A—C4A—C41A	121.3 (3)
C3—C2—C21	112.8 (3)	C4A—C5A—C6A	120.9 (3)
C1—C2—C3	118.4 (3)	N1A—C6A—C5A	119.4 (4)
C2—C3—C4	122.7 (3)	C3A—C2A—H2A	120.00
C3—C4—C5	118.8 (3)	N1A—C2A—H2A	120.00
Cl4—C4—C3	119.5 (3)	C2A—C3A—H3A	120.00
Cl4—C4—C5	121.7 (3)	C4A—C3A—H3A	120.00
Cl5—C5—C4	121.7 (3)	C6A—C5A—H5A	120.00
C4—C5—C6	119.4 (3)	C4A—C5A—H5A	120.00
Cl5—C5—C6	118.9 (2)	N1A—C6A—H6A	120.00
C1—C6—C5	122.4 (3)	C5A—C6A—H6A	120.00
O12—C11—C1	119.2 (3)	C4A—C41A—H42A	109.00
O11—C11—C1	118.7 (3)	C4A—C41A—H43A	109.00
O11—C11—O12	122.1 (3)	C4A—C41A—H41A	110.00
O21—C21—O22	122.3 (3)	H41A—C41A—H43A	110.00
O21—C21—C2	118.5 (3)	H42A—C41A—H43A	109.00
O22—C21—C2	119.2 (3)	H41A—C41A—H42A	109.00
			
C2A—N1A—C6A—C5A	0.7 (5)	C1—C2—C21—O22	169.9 (3)
C6A—N1A—C2A—C3A	0.2 (6)	C1—C2—C3—C4	0.1 (5)
C6—C1—C2—C21	−179.6 (3)	C2—C3—C4—C5	−1.2 (5)
C11—C1—C2—C21	0.3 (5)	C2—C3—C4—Cl4	180.0 (3)
C2—C1—C6—C5	0.7 (5)	C3—C4—C5—Cl5	−179.2 (2)
C11—C1—C6—C5	−179.1 (3)	Cl4—C4—C5—Cl5	−0.4 (4)
C2—C1—C11—O11	−173.2 (3)	Cl4—C4—C5—C6	−179.2 (2)
C2—C1—C11—O12	8.0 (5)	C3—C4—C5—C6	2.0 (5)
C6—C1—C11—O11	6.7 (4)	C4—C5—C6—C1	−1.8 (5)
C11—C1—C2—C3	180.0 (3)	Cl5—C5—C6—C1	179.4 (2)
C6—C1—C11—O12	−172.2 (3)	N1A—C2A—C3A—C4A	−0.3 (6)
C6—C1—C2—C3	0.1 (4)	C2A—C3A—C4A—C5A	−0.5 (5)
C21—C2—C3—C4	179.9 (3)	C2A—C3A—C4A—C41A	177.6 (3)
C1—C2—C21—O21	−11.1 (5)	C41A—C4A—C5A—C6A	−176.6 (3)
C3—C2—C21—O21	169.2 (3)	C3A—C4A—C5A—C6A	1.5 (5)
C3—C2—C21—O22	−9.8 (4)	C4A—C5A—C6A—N1A	−1.6 (5)

### Hydrogen-bond geometry (Å, °)

**Table:**

*D*—H···*A*	*D*—H	H···*A*	*D*···*A*	*D*—H···*A*
N1A—H1A···O1W	0.85 (5)	1.82 (5)	2.663 (5)	170 (5)
O1W—H11W···O21	0.79 (5)	2.02 (5)	2.793 (4)	168 (5)
O1W—H12W···O11^i^	0.78 (5)	2.03 (5)	2.806 (4)	170 (4)
O21—H21···O12	1.00 (6)	1.38 (6)	2.376 (4)	180 (8)
C2A—H2A···O12^i^	0.93	2.59	3.243 (5)	128
C2A—H2A···O12^ii^	0.93	2.54	3.147 (5)	123
C6A—H6A···O22	0.93	2.30	3.181 (5)	158

Symmetry codes: (i) *x*+1/2, −*y*+1/2, *z*−1/2; (ii) *x*+3/2, −*y*+1/2, *z*−1/2.
